# Genetic variants linked to type 2 diabetes in *CDKN1B* and *TCF7L2* influence survival outcomes in metastatic colorectal cancer

**DOI:** 10.1002/ijc.70035

**Published:** 2025-07-14

**Authors:** Raffaella Ruggiero, Alessandro Ottaiano, Madhura Tathode, Roberto Sirica, Annabella Di Mauro, Monica Ianniello, Nadia Petrillo, Massimiliano Berretta, Silvia Zappavigna, Amalia Luce, Michele Caraglia, Giovanni Savarese

**Affiliations:** ^1^ AMES, Centro Polidiagnostico Strumentale srl Casalnuovo Di Napoli Italy; ^2^ IRCCS “G. Pascale” Istituto Nazionale Tumori di Napoli Naples Italy; ^3^ Department of Precision Medicine University of Campania “L. Vanvitelli” Naples Italy; ^4^ Department of Clinical and Experimental Medicine University of Messina Messina Italy

**Keywords:** *CKN1B*, gene polymorphisms, metastatic colon cancer, prognosis, *TCF7L2*, type 2 diabetes

## Abstract

Evidence suggests that metastatic colorectal cancer patients with type 2 diabetes (T2D) experience a poorer prognosis in contrast to their non‐diabetic counterparts. Considering the multifactorial genetic nature of colon cancer development, we examined whether gene polymorphisms associated with T2D could affect the clinical outcome of metastatic colon cancer. Using in silico analysis, we evaluated gene variants linked to both T2D and colon cancer utilizing data from The Cancer Genome Atlas (TCGA). Subsequently, we assessed the prognostic relevance of polymorphisms in *CCND2*, *CDKN1B*, *CDKN2A*, *CDKN2B*, *EML4*, *HNF1A*, *ID3*, *IGF1*, *IGF1R*, *IGF2*, *INHBA*, *INSR*, *IRS1*, *IRS2*, and *TCF7L2* in a cohort of 99 consecutive metastatic non‐diabetic colon cancer patients with favorable clinical conditions. Primary colon cancer DNA was sequenced using the TruSight Oncology 500 kit, followed by sequencing on an Illumina NovaSeq 6000 platform. Notably, patients carrying the *CDKN1B* p.V109G and *TCF7L2* p.P370R polymorphisms exhibited significantly shorter median survivals compared to wild‐type counterparts, with adjusted hazard ratios (covariates: age, gender, metastatic extent, *RAS*/*BRAF* mutations, and response to therapy) of 2.28 (95% CI: 1.18–4.41) and 4.45 (95% CI: 1.26–15.70), respectively. Our findings provide scientific evidence of T2D genetic polymorphisms' involvement in determining the aggressiveness of metastatic colon cancer, identifying *CDKN1B* p.V109G and *TCF7L2* p.P370R as novel unfavorable prognostic markers.

## Graphical abstract

What's new?

Multiple genetic polymorphisms characteristic of type 2 diabetes (T2D) have been associated with unfavorable prognostic outcomes in individuals with both T2D and metastatic colorectal cancer. This study evaluated the impact of T2D genetic polymorphisms on metastatic colon cancer prognosis in a cohort of non‐diabetic patients. The results show that genetic variants traditionally implicated in T2D may influence the clinical trajectory of metastatic colorectal cancer independently of the clinical presence of T2D. *CDKN1B* p.V109G and *TCF7L2* p.P370R were identified as novel unfavorable prognostic markers. These findings illustrate the importance of genetic profiling in understanding disease behavior and optimizing patient management strategies.

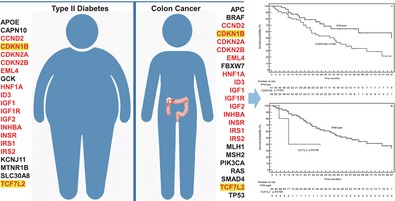

**Abbreviations**
ACMG: American College of Medical Genetics and Genomics
AMP: Association for Molecular Pathology
APC: Adenomatous Polyposis Coli
cBioPortal: Cancer Genomics Portal
CBMDB: Cancer Biomarker Database
CCND2: Cyclin D2
CDKN1B: Cyclin Dependent Kinase Inhibitor 1B
CDKN2A: Cyclin Dependent Kinase Inhibitor 2A
CDKN2B: Cyclin Dependent Kinase Inhibitor 2B
CDKs: Cyclin‐Dependent Kinases
CI: Confidence Intervals
CIViC: Clinical Interpretation of Variants in Cancer
ClinVar: Clinical Variants Database
COSMIC: Catalogue Of Somatic Mutations In Cancer
CRC: Colorectal Cancer
dbNSFP: Database for Nonsynonymous SNPs' Functional Predictions
DC: Disease Control
ECOG: Eastern Cooperative Oncology Group
EML4: Echinoderm Microtubule Associated Protein Like 4
ESMO: European Society for Medical Oncology
FFPE: Formalin‐Fixed Paraffin‐Embedded
G: Grading (tumor grade)
GENCODE: Genome Annotation Project Consortium
GEPIA2: Gene Expression Profiling Interactive Analysis version 2
GWAS: Genome‐Wide Association Study
HNF1A: Hepatocyte Nuclear Factor 1 Alpha
HRs: Hazard Ratios
ICGC‐PCAWG: International Cancer GenomeConsortium‐– Pan‐Cancer Analysis of Whole Genomes
ID3: Inhibitor of DNA Binding 3
IGF1: Insulin‐like Growth Factor 1
IGF1R: Insulin‐like Growth Factor 1 Receptor
IGF2: Insulin‐like Growth Factor 2
INHBA: Inhibin Subunit Beta A
INSR: Insulin Receptor
IRB: Institutional Review Board
IRS1: Insulin Receptor Substrate 1
IRS2: Insulin Receptor Substrate 2
KRAS: Kirsten Rat Sarcoma Viral Oncogene Homolog
Mb: Megabase
MedCalc®: Statistical Software for Biomedical Research
MSI: Microsatellite Instability
MSI‐H: Microsatellite Instability‐High
MSS: Microsatellite Stable
NGS: Next Generation Sequencing
OMIM: Online Mendelian Inheritance in Man
OncoScore: Oncogene Ranking Score Based on Text Mining
Orphanet: Rare Disease and Orphan Drug Database
OS: Overall Survival
PFS: Progression‐Free Survival
pN: Pathologic Regional Lymph Node Status
PS: Performance Status
pT: Pathologic Tumor Size/Extent
RECIST: Response Evaluation Criteria in Solid Tumors
SMAD4: Mothers Against Decapentaplegic Homolog 4
SNVs: Single Nucleotide Variants
T2D: Type 2 Diabetes
TCF7L2: Transcription Factor 7 Like 2
TCGA‐COAD: The Cancer Genome Atlas – Colon Adenocarcinoma
TMB: Tumor Mutational Burden
TP53: Tumor Protein p53
UALCAN: University of Alabama at Birmingham Cancer Data Analysis Portal
Wnt: Wingless/Integrated (signaling pathway)

## 1 INTRODUCTION

Colorectal cancer (CRC) is one of the most common malignancies worldwide, accounting for over 1 million new cases and 600,000 deaths annually.^1^, ^2^ Despite significant advances in early detection and treatment, metastatic CRC remains a significant cause of morbidity and mortality, with a 5‐year survival rate of only 10–15%.^3^, ^4^ CRC is a complex and multifactorial disease, with genetic, environmental, and lifestyle factors contributing to its development and progression. The Vogelstein model proposes that most of CRC arises from the accumulation of mutations in key driver genes over time, including *APC*, *KRAS*, *TP53*, and *SMAD4*, leading to dysregulation of the Wnt signaling pathway, cell proliferation, and apoptosis.^5^, ^6^ However, recent studies have suggested that CRC is a multi‐genic disease, with multiple genes and pathways involved in its pathogenesis and progression.^7^

The identification of genes contributing to CRC progression is pivotal for enhancing the diagnosis, prognosis, and therapeutic interventions for this malignancy. Notably, several genes are implicated in both type 2 diabetes (T2D), a widely prevalent condition, and CRC. These genes include *CCND2, CDKN1B, CDKN2A, CDKN2B, EML4, HNF1A, ID3, IGF1, IGF1R, IGF2, INHBA, INSR, IRS1, IRS2*, and *TCF7L2*.^8^ In fact, these genes exert significant influence on the regulation of glucose metabolism, insulin resistance, and the pathogenesis of T2D.^9^, ^10^, ^11^, ^12^ However, they also play integral roles in governing cellular proliferation, differentiation, and survival, thus intricately participating in diverse mechanisms of CRC initiation and progression. *CCND2* encodes cyclin D2, a pivotal regulator of the cell cycle, and has been demonstrated to contribute to CRC progression.^13^ *CDKN1B* and *CDKN2A*/*B* encode cyclin‐dependent kinase inhibitors, and their decreased expression has been correlated with the biological aggressiveness of CRC.^14^, ^15^, ^16^ *EML4*, a fusion partner of the *ALK* oncogene, has been identified as a potential therapeutic target in metastatic CRC.^17^, ^18^ *HNF1A* encodes hepatocyte nuclear factor 1 alpha, a transcription factor that governs glucose metabolism, and has been implicated in CRC progression.^19^ *ID3* encodes a helix–loop–helix transcription factor that is upregulated in CRC and fosters CRC‐initiating cells.^20^ *IGF1*, *IGF1R*, and *IGF2* encode insulin‐like growth factor 1, its receptor, and its ligand, respectively, and have been linked to various aspects of CRC development and progression.^21^, ^22^ *INHBA* encodes inhibin beta A, a member of the transforming growth factor‐beta superfamily, and has been shown to be functional and upregulated in CRC.^23^ *INSR* and *IRS1*/*2* encode insulin receptor and insulin receptor substrate proteins, respectively, and have been implicated in CRC progression and treatment sensitivity.^24^, ^25^, ^26^ *TCF7L2* encodes transcription factor 7‐like 2, a critical regulator of Wnt signaling, and has been associated with both CRC risk and progression.^27^, ^28^ Furthermore, metastatic CRC patients with T2D have a poorer prognosis compared to non‐diabetic patients. Additionally, multiple genetic polymorphisms characteristic of T2D have been associated with unfavorable prognostic outcomes in individuals with both T2D and metastatic CRC.^29^

In the present study, we have evaluated the impact of T2D genetic polymorphisms on metastatic colon cancer prognosis independently from the clinical presence of T2D in a cohort of non‐diabetic patients.

## 2 MATERIALS AND METHODS

### 2.1 Patients' selection and clinical management

The prognostic role of *CCND2*, *CDKN1B*, *CDKN2A*, *CDKN2B*, *EML4*, *HNF1A*, *ID3*, *IGF1*, *IGF1R*, *IGF2*, *INHBA*, *INSR*, *IRS1*, *IRS2*, and *TCF7L2* polymorphisms was investigated in a cohort of 99 metastatic colorectal cancer (mCRC) patients who sought evaluation at the AMES Center between 2018 and 2024 for DNA sequencing of their tumor tissue. Patients received treatment according to the European Society for Medical Oncology (ESMO) guidelines.^30^ All patients had an ECOG Performance Status (PS) <2, a cachexia risk <1,^31^ and a life expectancy >3 months. Moreover, only patients diagnosed with colon cancer were included in the analysis, given the well‐established clinical, biological, and molecular differences between colon and rectal cancers. Patients with type 2 diabetes (T2D), diagnosed according to the American Diabetes Association criteria,^32^, ^33^ were excluded. Treatment responses to first‐line therapies were assessed using the Response Evaluation Criteria in Solid Tumors (RECIST) v1.1.^34^

### 2.2 DNA sequencing

DNA sequencing was conducted on formalin‐fixed, paraffin‐embedded (FFPE) tissue specimens obtained from primary colon cancer samples. Prior to sequencing, tumor cells were microdissected under morphological guidance to ensure sample purity. DNA isolation was carried out using the MGF03‐Genomic DNA FFPE One‐Step Kit, following the manufacturer's protocol (MagCore Diatech). The quality of DNA was evaluated in triplicate using the FFPE QC Kit, also following the manufacturer's protocol (Illumina, San Diego). The TruSight Oncology 500 kit was used for preparing libraries, which targets the analysis of 523 genes related to cancer (the list is reported in Data S1). This assay detects small nucleotide variants (SNVs), indels, splice variants, copy number variants, fusions, and immunotherapy biomarkers such as tumor mutational burden (TMB) and microsatellite instability (MSI). Sequencing was performed on an Illumina NovaSeq 6000 (San Diego) platform. TMB was measured according to Chalmers et al.^35^ counting all coding, somatic base substitutions, and indels in the targeted regions, including synonymous alterations. The algorithms for “variant calling” and “TMB calculation” were independent; in this way, the number of coding variants cannot be derived from TMB and *vice versa* (see Manufacturer Instructions at https://emea.support.illumina.com/). The size of the targeted (coding) genomic region was 1.9 Mb. MSI, as a phenotype of clinical significance in CRCs, is the result of impaired DNA mismatch repair. The sequencing coverage and quality statistics of each sample are summarized in Table S1. A highly accurate exome‐based predictive model for the MSI phenotype was used. This model is based on a statistical MSI classifier from somatic mutation profiles that separates MSI‐H (MSI‐high) from MSS (MS stable) tumors. The MSI classifier was trained using 999 exome‐sequenced TCGA tumor samples with known MSI status (i.e., assayed from mononucleotide markers) and obtained a positive predictive value of 98.9% and a negative predictive value of 98.8% on an independent test set of 427 samples.

### 2.3 Phenolyzer analysis

Phenolyzer is a powerful computational tool that integrates multiple sources of genomic and phenotypic data to prioritize genes and variants implicated in the pathogenesis of a given disease. It interrogates the latest knowledge available, including protein–protein interactions, shared biological pathways, gene families, and gene–gene transcriptional regulation. It incorporates several gene‐disease databases, including OMIM, Orphanet, ClinVar, Gene Reviews, and GWAS Catalog. A more detailed explanation of its methodology can be found in Yang et al.^36^ In the context of this study, the tool was used to integrate information from multiple data sources, including gene expression profiles and functional annotations, to identify (among the 15 genes of interest: *CCND2*, *CDKN1B*, *CDKN2A*, *CDKN2B*, *EML4*, *HNF1A*, *ID3*, *IGF1*, *IGF1R*, *IGF2*, *INHBA*, *INSR*, *IRS1*, *IRS2*, and *TCF7L2*) the candidate genes that are likely to play a major role in influencing the clinical behavior of metastatic colon cancer. Results are presented using a scoring system and a network visualization tool that displays gene‐cancer and gene–gene relationships, providing a comprehensive overview of the interactional context. The legend for the network visualization image of Phenolyzer based on specific interactional contexts is reported in Figure S1.

### 2.4 Bioinformatics analysis and data presentation

The reported sequencing results and subsequent analyses were centered on the following genes: *CCND2*, *CDKN1B*, *CDKN2A*, *CDKN2B*, *EML4*, *HNF1A*, *ID3*, *IGF1*, *IGF1R*, *IGF2*, *INHBA*, *INSR*, *IRS1*, *IRS2*, and *TCF7L2*. Comparative expression profiling analysis was performed to study the expression of genes in TCGA‐COAD primary tumor samples relative to the control using the online UALCAN program [https://ualcan.path.uab.edu/index.html].^37^ UALCAN uses the Student's t‐test to determine the statistical significance between the control and tumor groups to conduct a comparative expression profiling analysis. The gene expression is considered significant if the statistical significance value is <0.05. Additionally, the list of protein‐coding genes was assessed for its association with patient survival in TCGA‐COAD datasets by deriving Kaplan–Meier (KM) plots based on its expression and mutation status. Online GEPIA2 [http://gepia2.cancer-pku.cn/#index]^38^ and cBioPortal (https://www.cbioportal.org/)^39^ program were used to derive Kaplan–Meier (KM) plots based on the expression and mutation status of a gene, respectively.

Illumina TruSight Oncology 500 bioinformatics pipeline was applied to analyze sequencing results. A median of 117 million reads was generated for each sample, and the coverage in the target region was above the manufacturer's suggested threshold of 150×. Sequence data were aligned to the human reference genome GRCh37 (http://www.ncbi.nlm.nih.gov/projects/genome/assembly/grc/ human/index.shtml) using the Burrows–Wheeler Aligner with default parameters.^40^ Both population‐ and cancer‐specific variants were intersected with GENCODE, dbNSFP, ICGC‐PCAWG, COSMIC, 1000Genomes, ClinVar, CancerMine, OncoScore, CIViC, CBMDB databases to assess the clinical significance of the found variants. Variants were filtered with unmatched normal datasets and removed if the global minor allele frequency was <1%. The prioritization of variants was done according to a four‐tiered structure, adopting the joint consensus recommendation by AMP/ACMG.^41^ Variants of strong clinical significance in cancer were defined considering items with strongest evidence levels in the database for (i) clinical interpretations of variants in cancer (CIViC, civicdb.org) and (ii) Cancer Biomarkers (cancergenomeinterpreter.org/biomarkers).

The prognostic impact of the genes polymorphisms on overall survival (OS) of metastatic colon cancer patients was studied. The OS was assessed from the diagnosis of metastatic disease until death from CRC (cancer‐specific survival). Progression‐free survival (PFS) was not included as a study objective because treatments and radiologic evaluations were heterogeneous, not centralized, and the vital status is a more reliable and solid outcome to report and analyze.

In univariate and multivariate analyses, putative prognostic factors (covariates) were dichotomized: age (≤70 vs. >70 years), gender (male vs. female), metastatic involvement (one site vs. more than one site), *RAS*/*BRAF* mutations (presence vs. wild‐type), and response to first‐line therapy (disease control vs. no disease control). OS was generated through the Kaplan–Meier product limit method. Statistical significance at univariate analysis was evaluated with a two‐tailed log‐rank test. Multivariate analysis was applied to study prognostic interactions between OS and covariates; the test was based on the Cox proportional‐hazards regression model. The estimates of the survival probability according to different covariates were expressed through the HRs (Hazard Ratios) which are the risk of event (death), at any time, for a patient with the risk factor present compared to a patient with the risk factor absent (given both patients identical for all other covariates). HRs were reported in any analyses with 95% confidence intervals (CI). Statistical analyses were done using the Excel software and MedCalc® version 20.112. Associations between genes polymorphisms and response to therapy were depicted through contingency tables and evaluated by χ^2^ test. *p* < 0.05 was considered statistically significant.

## 3 RESULTS

### 3.1 Genes prioritization

**FIGURE 1:**
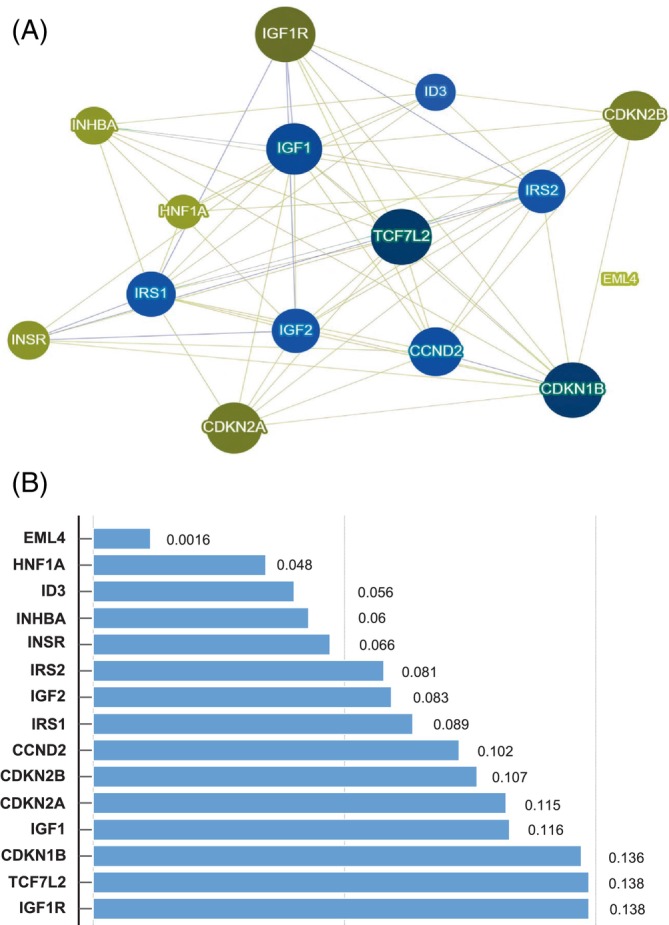
Phenolyzer systematically examines key gene‐disease databases (see Section 2) to prioritize genes by considering current scientific knowledge such as shared biological pathways, gene families, gene–gene transcriptional regulation, and protein–protein interactions. The outcomes are presented through a network visualization image (A), and a score system visible at the end of each bar in the graph (B), offering readers an intuitive overview of the weighted interactions of the involved genes.

Genetic polymorphisms in genes associated with T2D, including *CCND2*, *CDKN1B*, *CDKN2A*, *CDKN2B*, *EML4*, *HNF1A*, *ID3*, *IGF1*, *IGF1R*, *IGF2*, *INHBA*, *INSR*, *IRS1*, *IRS2*, and *TCF7L2*, have been implicated in promoting a more aggressive neoplastic phenotype in diabetic colon cancer patients.^29^ Given this potential link, we sought to evaluate the impact of these T2D‐related gene polymorphisms in metastatic colon cancer patients independently from the presence of T2D. To establish a preliminary ranking of these genes based on available public data, we interrogated TCGA‐COAD. Differential expression analysis between normal and tumor tissues revealed that the vast majority of these genes exhibit significant alterations in cancer (Table 1). This indirectly confirmed the potential involvement of all the genes found altered in T2D in colon cancer development. Furthermore, survival analyses based on gene expression (TCGA & GTEx profile) and mutational status (TCGA COAD & Pan‐cancer datasets) for colon adenocarcinoma identified *CDKN2A*, *INHBA* increased expression, and *CDKN2A* and *TCF7L2* mutations as predictors of bad prognosis in metastatic patients (Table 1). These data confirm, in large and public datasets, that genes involved in the pathogenesis of T2D contribute to colon cancer. Analysis through Phenolyzer (see Section 2) highlights that among these genes, *IGF1R*, *TCF7L2*, and *CDKN1B* are the most significant and interrelated (Figure 1). Interestingly, *TCF7L2* gene was found to be very significantly associated with TCGA‐COAD patients in OS analysis as well as Phenolyzer analysis, emphasizing its crucial role in the progression of colon cancer. Table 2 presents the demographic and clinical features of our study cohort.

**TABLE 1:** Gene expression/mutation and prognostic implications in TCGA colon adenocarcinoma dataset (TCGA‐COAD).

Gene	TPM (Primary tumor vs. Normal)	Statistical significance (Primary tumor vs Normal)	Expression status (Primary tumor vs Normal)	Log rank *p*‐value* (for altered expression)	Interpretation	Log rank *p*‐value* (for gene mutation)	Interpretation
*CCND2*	38.773	3.20E‐09	Significantly upregulated	0.73	NS	0.72	NS
*CDKN1B*	57.847	1.28E‐09	Significantly upregulated	0.19	NS	0.96	NS
*CDKN2A*	3.917	2.00E‐15	Significantly upregulated	0.01	The high expression worsens the prognosis.	0.05	The presence of mutations worsens the prognosis.
*CDKN2B*	5.046	1.63E‐12	Significantly downregulated	0.38	NS	0.14	NS
*EML4*	42.403	1.56E‐09	Significantly upregulated	0.54	NS	0.27	NS
*HNF1A*	20.186	2.29E‐14	Significantly upregulated	0.79	NS	0.64	NS
*ID3*	85.424	1.20E‐08	Significantly downregulated	0.52	NS	0.86	NS
*IGF1*	0.531	3.51E‐07	Significantly downregulated	0.91	NS	0.13	NS
*IGF1R*	7.661	4.43E‐02	Significantly upregulated	0.08	NS	0.16	NS
*IGF2*	7.165	2.24E‐05	Significantly downregulated	0.45	NS	0.23	NS
*INHBA*	5.581	1.62E‐12	Significantly upregulated	0.04	The high expression worsens the prognosis.	0.19	NS
*INSR*	11.642	5.63E‐07	Significantly downregulated	0.71	NS	0.79	NS
*IRS1*	6.638	2.39E‐06	Significantly upregulated	0.39	NS	0.41	NS
*IRS2*	13.73	1.65E‐12	Significantly upregulated	0.59	NS	0.52	NS
*TCF7L2*	23.216	3.31E‐10	Significantly downregulated	0.54	NS	0.03	The presence of mutations improves the prognosis.

*Note*: Comparison criteria are indicated between square brackets. *Significance of the difference in survival observed in the Kaplan–Meier plots.

Abbreviations: NS, not significant; TPM, transcripts per million.

**TABLE 2:** Clinico‐pathological characteristics of analysed patients.

Variable	No.	%
Age		
Median, range (year)	63, 27–82	
Gender		
Male	64	64.6
Female	35	35.4
Side		
Left	69	69.7
Right	30	30.3
Grading		
G1	7	7.1
G2/G3	92	92.9
pT		
1/2	13	13.1
3	52	52.5
4	12	12.1
Unknown	22	22.2
pN		
0	29	29.3
1	30	30.3
2	18	18.2
Unknown	22	22.2
No. of metastatic sites		
1	74	74.7
≥2	25	25.3
*RAS (K or N)*		
Mutated	45	45.5
Wild‐type	54	54.5
*BRAF*		
Mutated	7	7.1
Wild‐type	92	92.9
MSS		
Stable	92	92.9
Unstable	7	7.1
Previous adjuvant therapy		
Yes	42	42.4
No	57	57.6
Response to first‐line chemotherapy		
DC	78	78.8
No DC	21	21.2

The median age of the enrolled patients was 63 years, with an age ranging from 27 to 82 years. Gender distribution skewed towards males (64.6% males and 35.4% females). The predominant site of tumors was the left side (69.7%). Histopathological grading indicated that the majority of tumors were categorized as G2/G3 (92.9%), with a minority classified as G1 (7.1%). Pathological staging unveiled a variety of primary tumor (pT) stages: pT1/2 (13.1%), pT3 (52.5%), pT4 (12.1%), and an unknown stage in 22.2% of cases in which primary tumor resection was not performed. Local lymph node involvement (pN) ranged from pN0 (29.3%) to pN2 (18.2%), with 22.2% of cases that did not undergo surgery categorized as unknown. In most cases, metastases at onset were confined to a single site (74.7%), while a smaller proportion exhibited metastases in more than two sites (25.3%). Molecular analysis unveiled a *RAS* gene mutation in 45.5% of cases, while the remaining 54.5% were wild‐type. *BRAF* mutations were infrequent, with 7.1% of cases harboring mutations, and the vast majority being wild‐type (92.9%). Microsatellite stability (MSS) analysis revealed a predominance of stable status (92.9%) versus unstable ones (7.1%). Previous adjuvant therapy was reported in 42.4% of cases, while 57.6% of the remaining patients did not receive adjuvant treatments. Regarding the response to first‐line chemotherapy, disease control (DC) was achieved in most cases (78.8%).

### 3.2 Impact of T2D gene polymorphisms on the clinical behavior of metastatic colon cancer

**FIGURE 2:**
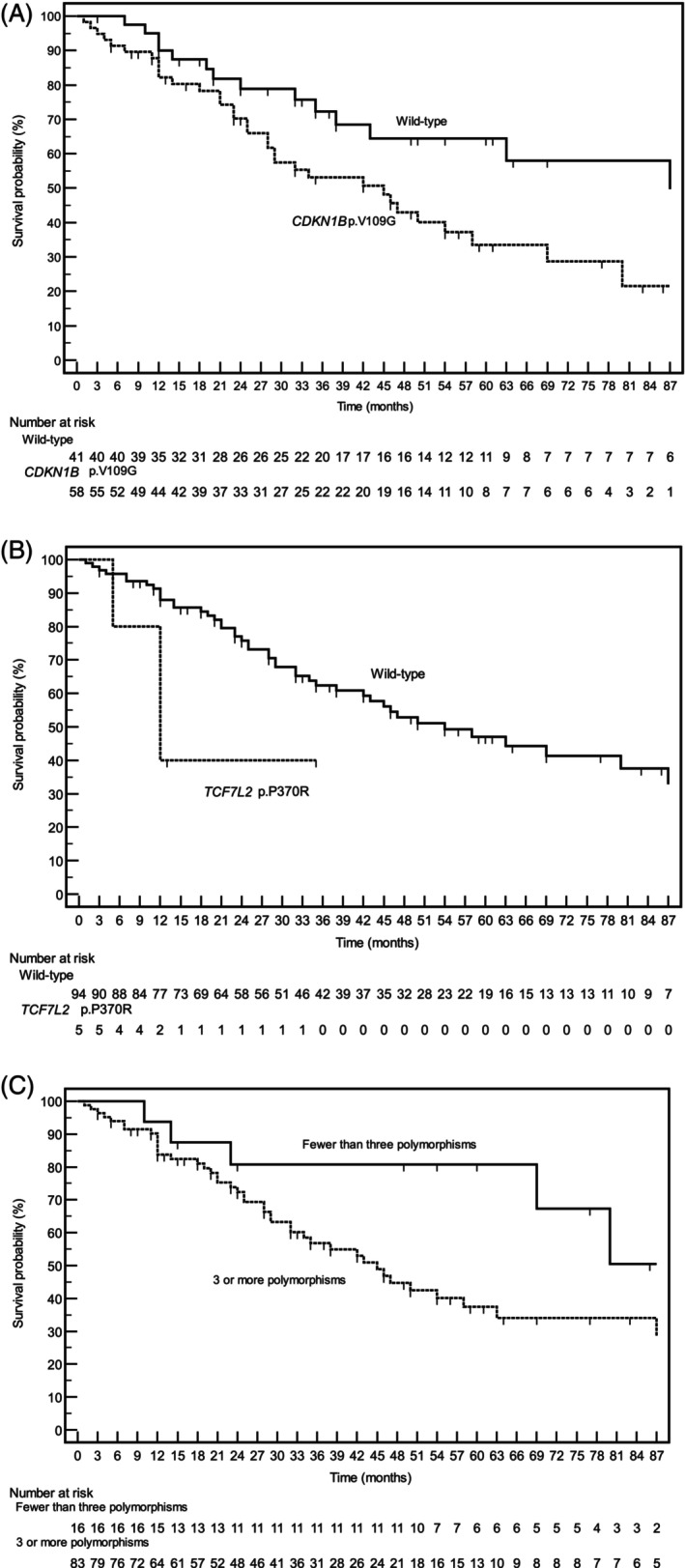
Kaplan–Meier survival curves, stratified by gene polymorphisms significantly associated with overall survival in the analyzed cohorts, are shown. The corresponding hazard ratios and *p*‐values are reported in Table 3. (A) Overall survival in patients with the CDKN1B wild‐type allele compared to those carrying the p.V109G variant. (B) Prognostic trends in patients with the TCF7L2 wild‐type allele versus those harboring the p.P370R variant. (C) Survival outcomes in patients carrying more than three polymorphisms versus those with three or fewer.

All the tumour tissues derived from the enrolled patients were sequenced through NGS as described in the Section 2. We selected gene polymorphisms linked to an amino acid substitution within a coding region, which were detected in a minimum of 5 and a maximum of 94 out of the 99 enrolled patients. These criteria were applied to ensure both clinical relevance and analytical feasibility. Polymorphisms, along with their frequencies, are reported in the Table S2. This approach revealed 10 polymorphisms with potential clinical relevance. After a median follow‐up of 42 months, there were 50 deaths related to metastatic colon cancer. Among the examined gene polymorphisms, two exhibited significant associations with prognosis. Notably, patients harboring the *CDKN1B* p.V109G polymorphism demonstrated a median overall survival (mOS) of 45.0 months vs. the 87.0 months observed in those with the wild‐type variant (HR: 2.13; 95% CI: 1.19–3.79; *p* = 0.0102). Additionally, the *TCF7L2* p.P370R variant was associated with a notably lower mOS of 19.8 months compared to 61.6 months in wild‐type carriers (HR: 8.13; 95% CI: 1.09–60.60; *p* = 0.0409). Moreover, the coexistence of more than three polymorphisms was linked to a mOS of 45.0 months, significantly differing from patients with fewer than three polymorphisms (mOS not reached; HR: 2.13; 95% CI: 1.08–4.18; *p* = 0.0278) (Table 3). Kaplan–Meier survival curves are depicted in Figure 2. These findings underscore the prognostic relevance of specific gene polymorphisms in metastatic colon cancer patients and suggest that the co‐presence of multiple polymorphisms (regardless of type) may have an additive detrimental effect on prognosis.

**TABLE 3:** Impact of selected T2D polymorphisms on metastatic colon cancer prognosis.

Polymorphisms	Dichotomization	Reference SNP	mOS (months)	No. of events/patients	HR	95% CI	*P* at Log Rank test
*CDKN1B* p.V109G	vs WT	rs2066827	45.0 (vs 87.0)	34/58 (vs 16/41)	2.13	1.19–3.79	0.0102
*CDKN2A* p.A148T	vs WT	rs3731249	47.0 (vs 88.0)	3/10 (vs 47/89)	0.53	0.22–1.25	0.1514
*EML4* p.K409R	vs WT	rs not found	47.0 (vs 69.0)	25/49 (vs 25/50)	1.19	0.67–2.11	0.5322
*HNF1A* p.I27L	vs WT	rs1169288	45.0 (vs 80.0)	35/64 (vs 15/35)	1.62	0.90–2.90	0.1049
*HNF1A* p.S487N	vs WT	rs2464196	50.0 (vs 58.0)	27/52 (vs 23/47)	1.37	0.77–2.42	0.2728
*INSR* p.A2G	vs WT	rs7508518	54.0 (vs 47.0)	34/65 (vs 16/34)	0.81	0.43–1.52	0.5137
*INSR* p.V975M	vs WT	rs not found	54.0 (vs 50.0)	2/6 (vs 48/93)	1.13	0.25–5.12	0.8720
*IRS1* p.G971R	vs WT	rs1801278	63.0 (vs 47.0)	6/16 (vs 44/83)	0.59	0.29–1.20	0.1510
*IRS2* p.G1057D	vs WT	rs1805097	46.0 (vs 63.0)	27/53 (vs 23/46)	1.24	0.70–2.18	0.4507
*TCF7L2* p.P370R	vs WT	rs not found	19.8 (vs 61.6)	3/5 (vs 47/94)	8.13	1.09–60.60	0.0409
Coexistence of more than 3 polymorphisms	vs ≤3	NA	45.0 (vs NR)	45/83 (vs 5/16)	2.13	1.08–4.18	0.0278

Abbreviations: CI, confidence interval; HR, hazard ratio; mOS, median overall survival; mut: mutated; NA, not applicable; NR, not reached; WT, wild‐type.

To verify the independence of the prognostic effect from other clinical or biological variables, a multivariate analysis was performed for each previously identified element (*CDKN1B* p.V109G, *TCF7L2* p.P370R, coexistence of 3 polymorphisms). The covariates of the model included age (≤70 vs. >70 years), gender (male vs. female), metastatic involvement (one site vs. more than one site), *RAS*/*BRAF* mutations (presence vs. wild‐type), and response to first‐line therapy (disease control vs. no disease control). *CDKN1B* p.V109G and *TCF7L2* p.P370R remained independent prognostic factors in the multivariate model (Table 4).

**TABLE 4:** Multivariate analysis of prognostic power of selected polymorphisms in metastatic colon cancer.

Gene*	Dichotomization	HR	95% CI	*p* at multivariate
*CDKN1B*	p.V109G vs. WT	2.28	1.18–4.41	0.0137
*TCF7L2*	p.P370R vs. WT	4.45	1.26–15.70	0.0200
No. of polymorphisms	>3 vs. ≤3	2.26	0.85–6.04	0.0748

*Note*: *The analysis is adjusted for each gene based on age (≤70 vs. >70 years), gender (male vs. female), metastatic involvement (one site vs. more than one site), *RAS*/*BRAF* mutations (presence vs. wild‐type), and response to first‐line therapy (disease control vs. no disease control).

Abbreviations: CI, confidence interval; HR, hazard ratio; WT, wild‐type.

As some highlighted genes are involved in biological pathways affecting cell proliferation and survival, thereby modifying treatment response, we investigated whether they were associated with first‐line therapy sensitivity. No significant association was found between the presence of the polymorphism and disease control or tumor sidedness, as reported in Table S3.

## 4 DISCUSSION

In the present manuscript, we verified if genetic polymorphisms involved in the pathogenesis of T2D could be involved in the clinical aggressiveness of colon cancer.

It is evident that in recent years, the idea of the complexity of cancer genetics has been gaining ground, wherein not only the presence and activity of genes carrying clearly pathogenic mutations (*RAS*, *BRAF*, *p53*, etc.) play a role, but also the additive effect (“dose effect”) of genes that, even if not being driver oncogenes, modify their activity in favor of dysregulating cancer‐related effects (proliferation, differentiation, production of growth factors, etc.). The latter is a biological factor that additionally complicates the understanding of the mechanisms of tumor progression. The study of genes for which it is known their involvement in T2D, a condition associated with worse CRC prognosis, is a good model to deepen this scientific gap.

Firstly, we used prioritization and analysis tools to clearly identify the T2D‐associated genes most involved in the modification of colon carcinoma clinical outcome. Analysis of publicly available datasets confirmed in silico the dysregulation of gene expression in colon cancer and their involvement in influencing prognosis. However, these analyses may be coarse due to the considerable heterogeneity of the datasets. Therefore, they can be useful to generate hypotheses, but they must be validated in selected and well‐characterized patient series. Additionally, the use of Phenolyzer allowed us to further prioritize the genes based on their functional relevance to the phenotype of interest, considering the strength of the functional evidence supporting gene involvement. It is noteworthy, and we wish to emphasize it from the outset, that both in this initial analysis and in subsequent ones, the genes maintaining a significant role are *CDKN1B* and *TCF7L2*. This is a very intriguing finding that may provide a basis for additional studies and insights for in vitro experiments aimed at defining the role of both wild‐type and altered gene products in the near future.

Our investigation unveiled specific gene polymorphisms significantly influencing patient survival. Notably, individuals carrying the *CDKN1B* p.V109G polymorphism exhibited markedly shorter mOS compared to those with the wild‐type variant, underscoring its clinical significance. Similarly, the *TCF7L2* p.P370R variant was associated with a notably lower mOS, suggesting its potential involvement in driving colon cancer progression. These findings are supported by a biological and molecular rationale indicating the strong role of these two genes in the determination of cancer development and progression. In fact, *CDKN1B*, also known as cyclin‐dependent kinase inhibitor 1B, acts as a critical regulator of cell cycle progression, primarily functioning as a tumor suppressor by inhibiting cyclin‐dependent kinases (CDKs) and arresting cells in the G1 phase. The *CDKN1B* gene is located on chromosome 12p13.1, spanning approximately 12.3 kilobases, and consists of four exons and three introns.^42^, ^43^ *TCF7L2*, transcription factor 7‐like 2, belongs to the T‐cell factor/lymphoid enhancer factor (TCF/LEF) family of transcription factors. It plays a pivotal role in the Wnt signaling pathway, regulating gene expression involved in cell proliferation, differentiation, and development. The *TCF7L2* gene is situated on chromosome 10q25.2, spanning approximately 44.5 kilobases, and comprises 17 exons and 16 introns.^27^, ^44^, ^45^ Mutations in *CDKN1B* and *TCF7L2* have been implicated in the initiation and progression of various cancers.^42^, ^43^, ^44^, ^45^

Although it was statistically futile in this study to analyze the prognostic effect of both polymorphisms simultaneously as it can only improve, examining the prognostic impact of different combinations of genetic polymorphisms was also impractical due to the vast number of possible combinations. However, to explore the additive effect of polymorphisms, we assessed whether the presence of more than three polymorphisms had a prognostic impact. This was evident in univariate analysis but lost significance in multivariate analysis. Nevertheless, it is essential to note that multivariate analysis is not an absolute dogma nor the panacea of statistical inference and scientific validation of a prognostic factor. In other words, the biological and prognostic implications of a gene polymorphism that influences cell proliferation, particularly when it aligns with a pathogenic mutation such as *KRAS* or is prevalent in females, remain significant even if its statistical significance diminishes in multivariate analysis. Therefore, the interpretation of *p*‐values, especially in multivariate analysis, should be nuanced, and due respect should be given to the univariate hazard ratios, ensuring they are interpreted appropriately.

Investigation into the association between these genetic variations and response to first‐line therapy was also performed, revealing no significant correlations. This suggests that the prognostic impact of these polymorphisms is independent of response to treatment.

A major strength of our study is the clinical and methodological homogeneity of the patient cohort. Patients were carefully selected to minimize confounding factors related to general clinical conditions, ensuring that all participants were in good overall health. Additionally, all genetic assessments were conducted in the same laboratory using a standardized NGS assay, ensuring consistency in data acquisition. However, the limited sample size remains a notable limitation that should be acknowledged and considered when interpreting the findings.

A critical consideration, both a limitation of the present study and a springboard for future investigation, is the exclusion of patients with T2D. While this design choice minimized confounding by systemic metabolic alterations, it also precluded direct comparison of the prognostic impact of these polymorphisms in diabetic versus non‐diabetic patients with metastatic colon cancer. Including a dedicated T2D cohort in future studies would be instrumental in elucidating the interplay between metabolic dysregulation and genetic variation. In fact, key metabolic features of T2D, such as hyperinsulinemia, chronic low‐grade inflammation, and impaired glucose homeostasis, may modulate the functional effects of *CDKN1B* and *TCF7L2* variants. A prospective, stratified analysis of patients by T2D status, integrating metabolic profiling and glycemic indices, could uncover gene–environment interactions that refine prognostic modeling and risk stratification. Such insights would lay the foundation for personalized therapeutic strategies that incorporate both oncogenic drivers and metabolic determinants of tumor behavior.

Moreover, while our findings highlight the adverse prognostic role of these polymorphisms, their broader clinical implications merit deeper consideration. Not all patients with T2D and cancer exhibit poor outcomes, and the literature on this association remains inconsistent. A plausible explanation is that the presence or absence of specific genetic variants may contribute to the observed heterogeneity in survival among T2D metastatic colon cancer patients. Those with outcomes similar to non‐diabetic patients may, in fact, lack these high‐risk polymorphisms, potentially accounting for the discordant clinical evidence. This speculative but compelling hypothesis underscores the translational relevance of our results and supports the need for integrated molecular and metabolic profiling in future research.

## 5 CONCLUSIONS

In conclusion, our findings provide scientific evidence supporting the hypothesis that T2D genetic polymorphisms are involved in the aggressiveness of the disease in patients with metastatic colon cancer, shedding light on potential biomarkers for risk stratification and personalized treatment approaches in this clinical setting. These results underscore the importance of genetic profiling in understanding disease behavior and optimizing patient management strategies. Additional research with larger cohorts is required in order to validate these findings and explore their clinical implications comprehensively.

## AUTHOR CONTRIBUTIONS

**Raffaella Ruggiero:** Conceptualization; formal analysis; investigation; data curation; writing – original draft. **Alessandro Ottaiano:** Conceptualization; formal analysis; methodology; validation; writing – original draft. **Madhura Tathode:** Formal analysis; methodology; validation; writing – review and editing. **Roberto Sirica:** Formal analysis; methodology; software; writing – review and editing. **Annabella Di Mauro:** Validation; writing – original draft. **Monica Ianniello:** Writing – original draft; validation. **Nadia Petrillo:** Investigation; validation. **Massimiliano Berretta:** Validation; writing – review and editing. **Silvia Zappavigna:** Validation; writing – review and editing. **Amalia Luce:** Methodology; writing – review and editing. **Michele Caraglia:** Supervision; formal analysis; writing – review and editing. **Giovanni Savarese:** Conceptualization; supervision; resources; formal analysis; writing – original draft.

## FUNDING INFORMATION

This study was funded by the AMES Center.

## CONFLICT OF INTEREST STATEMENT

Roberto Sirica, Monica Ianniello, Raffaella Ruggiero, Nadia Petrillo, and Giovanni Savarese are affiliated with AMES, Centro Polidiagnostico Strumentale Srl, located in 80013 Naples, Italy. These activities were conducted independently and were unrelated to the submitted work. The remaining authors declare no conflicts of interest related to this study and affirm that the research was conducted without any commercial or financial relationships that could be perceived as potential conflicts of interest.

## ETHICS STATEMENT

This study was conducted in accordance with the Declaration of Helsinki. All patients provided written informed consent before undergoing genetic assessments. The study protocol was approved by the Institutional Review Board (IRB) of the AMES Center under protocol number 04CA/2024.

## Supporting information


**DATA S1.** Supporting information.

**TABLE S1.** Sequencing coverage and quality metrics for each sample.

**TABLE S2.**Polymorphisms identified in the analyzed genes, along with their frequencies.

## Data Availability

The data that support the findings of this study are available from the corresponding author upon reasonable request.
