# Supplementary material for: Genetic variants linked to type 2 diabetes in CDKN1B and TCF7L2 influence survival outcomes in metastatic colorectal cancer
Source: Int J Cancer. 2025 Jul 14;157(9):1853–63. doi: 10.1002/ijc.70035 (PMC12407037; doi:10.1002/ijc.70035)

Genetic variants linked to type 2 diabetes in *CDKN1B* and *TCF7L2* influence survival outcomes in metastatic colorectal cancer

Raffaella Ruggiero, Alessandro Ottaiano, Madhura Tathode, Roberto Sirica, Annabella Di Mauro, Monica Ianniello, Nadia Petrillo, Massimiliano Berretta, Silvia Zappavigna, Amalia Luce, Michele Caraglia, Giovanni Savarese

Table of contents

Supplementary materials & methods.......... 2
Supplementary tables.......... 4
Supplementary figures.......... 5

Supplementary materials & methods

List of genes analyzed using the TSO500 panel

ABL1, ABL2, ACVR1, ACVR1B, AKT1, AKT2, AKT3, ALK, ALOX12B, ANKRD11, ANKRD26, APC, AR, ARAF, ARFRP1, ARID1A, ARID1B, ARID2, ARID5B, ASXL1, ASXL2, ATM, ATR, ATRX, AURKA, AURKB, AXIN1, AXIN2, AXL, B2M, BAP1, BARD1, BBC3, BCL10, BCL2, BCL2L1, BCL2L11, BCL2L2, BCL6, BCOR, BCORL1, BCR, BIRC3, BLM, BMPR1A, BRAF, BRCA1, BRCA2, BRD4, BRIP1, BTG1, BTK, C11orf30, CALR, CARD11, CASP8, CBFB, CBL, CCND1, CCND2, CCND3, CCNE1, CD274, CD276, CD74, CD79A, CD79B, CDC73, CDH1, CDK12, CDK4, CDK6, CDK8, CDKN1A, CDKN1B, CDKN2A, CDKN2B, CDKN2C, CEBPA, CENPA, CHD2, CHD4, CHEK1, CHEK2, CIC, CREBBP, CRKL, CRLF2, CSF1R, CSF3R, CSNK1A1, CTCF, CTLA4, CTNNA1, CTNNB1, CUL3, CUX1, CXCR4, CYLD, DAXX, DCUN1D1, DDR2, DDX41, DHX15, DICER1, DIS3, DNAJB1, DNMT1, DNMT3A, DNMT3B, DOT1L, E2F3, EED, EGFL7, EGFR, EIF1AX, EIF4A2, EIF4E, EML4, EP300, EPCAM, EPHA3, EPHA5, EPHA7, EPHB1, ERBB2, ERBB3, ERBB4, ERCC1, ERCC2, ERCC3, ERCC4, ERCC5, ERG, ERRFI1, ESR1, ETS1, ETV1, ETV4, ETV5, ETV6, EWSR1, EZH2, FAM123B, FAM175A, FAM46C, FANCA, FANCC, FANCD2, FANCE, FANCF, FANCG, FANCI, FANCL, FAS, FAT1, FBXW7, FGF1, FGF10, FGF14, FGF19, FGF2, FGF23, FGF3, FGF4, FGF5, FGF6, FGF7, FGF8, FGF9, FGFR1, FGFR2, FGFR3, FGFR4, FH, FLCN, FLI1, FLT1, FLT3, FLT4, FOXA1, FOXL2, FOXO1, FOXP1, FRS2, FUBP1, FYN, GABRA6, GATA1, GATA2, GATA3, GATA4, GATA6, GID4, GLI1, GNA11, GNA13, GNAQ, GNAS, GPR124, GPS2, GREM1, GRIN2A, GRM3, GSK3B, H3F3A, H3F3B, H3F3C, HGF, HIST1H1C, HIST1H2BD, HIST1H3A, HIST1H3B, HIST1H3C, HIST1H3E, HIST1H3F, HIST1H3G, HIST1H3H, HIST1H3I, HIST1H3J, HIST2H3A, HIST2H3C, HIST2H3D, HIST3H3, HLA-A, HLA-B, HLA-C, HNF1A, HNRNPK, HOXB13, HRAS, HSD3B1, HSP90AA1, ICOSLG, ID3, IDH1, IDH2, IFNGR1, IGF1, IGF1R, IGF2, IKBKE, IKZF1, IL10, IL7R, INHA, INHBA, INPP4A, INPP4B, INSR, IRF2, IRF4, IRS1, IRS2, JAK1, JAK2, JAK3, JUN, KAT6A, KDM5A, KDM5C, KDM6A, KDR, KEAP1, KEL, KIF5B, KIT, KLF4, KLHL6, KMT2B, KMT2C, KMT2D, KRAS, LAMP1, LATS1, LATS2, LMO1, LRP1B, LYN, LZTR1, MAGI2, MALT1, MAP2K1, MAP2K2, MAP2K4, MAP3K1, MAP3K13, MAP3K14, MAP3K4, MAPK1, MAPK3, MAX, MCL1, MDC1, MDM2, MDM4, MED12, MEF2B, MEN1, MET, MGA, MITF, MLH1, MLL, MLLT3, MPL, MRE11A, MSH2, MSH3, MSH6, MST1, MST1R, MTOR, MUTYH, MYB, MYC, MYCL1, MYCN, MYD88, MYOD1, NAB2, NBN, NCOA3, NCOR1, NEGR1, NF1, NF2, NFE2L2, NFKBIA, NKX2-1, NKX3-1, NOTCH1, NOTCH2, NOTCH3, NOTCH4, NPM1, NRAS, NRG1, NSD1, NTRK1, NTRK2, NTRK3, NUP93, NUTM1, PAK1, PAK3, PAK7, PALB2, PARK2, PARP1, PAX3, PAX5, PAX7, PAX8, PBRM1, PDCD1, PDCD1LG2, PDGFRA, PDGFRB, PDK1, PDPK1, PGR, PHF6, PHOX2B, PIK3C2B, PIK3C2G, PIK3C3, PIK3CA, PIK3CB, PIK3CD, PIK3CG, PIK3R1, PIK3R2, PIK3R3, PIM1, PLCG2, PLK2, PMAIP1, PMS1, PMS2, PNRC1, POLD1, POLE,

PPARG, PPM1D, PPP2R1A, PPP2R2A, PPP6C, PRDM1, PREX2, PRKAR1A, PRKCI, PRKDC, PRSS8, PTCH1, PTEN, PTPN11, PTPRD, PTPRS, PTPRT, QKI, RAB35, RAC1, RAD21, RAD50, RAD51, RAD51B, RAD51C, RAD51D, RAD52, RAD54L, RAF1, RANBP2, RARA, RASA1, RB1, RBM10, RECQL4, REL, RET, RFWD2, RHEB, RHOA, RICTOR, RIT1, RNF43, ROS1, RPS6KA4, RPS6KB1, RPS6KB2, RPTOR, RUNX1, RUNX1T1, RYBP, SDHA, SDHAF2, SDHB, SDHC, SDHD, SETBP1, SETD2, SF3B1, SH2B3, SH2D1A, SHQ1, SLIT2, SLX4, SMAD2, SMAD3, SMAD4, SMARCA4, SMARCB1, SMARCD1, SMC1A, SMC3, SMO, SNCAIP, SOCS1, SOX10, SOX17, SOX2, SOX9, SPEN, SPOP, SPTA1, SRC, SRSF2, STAG1, STAG2, STAT3, STAT4, STAT5A, STAT5B, STK11, STK40, SUFU, SUZ12, SYK, TAF1, TBX3, TCEB1, TCF3, TCF7L2, TERC, TERT, TET1, TET2, TFE3, TFRC, TGFBR1, TGFBR2, TMEM127, TMPRSS2, TNFAIP3, TNFRSF14, TOP1, TOP2A, TP53, TP63, TRAF2, TRAF7, TSC1, TSC2, TSHR, U2AF1, VEGFA, VHL, VTCN1, WISP3, WT1, XIAP, XPO1, XRCC2, YAP1, YES1, ZBTB2, ZBTB7A, ZFHX3, ZNF217, ZNF703, ZRSR2.

Supplementary tables

Table S1

Sequencing coverage and quality metrics for each sample are provided in a separate Excel file (Table S1).

Table S2

Polymorphisms identified in the analyzed genes, along with their frequencies, are reported in a separate Excel file (Table S2).

Table S3

Associations between genes polymorphism, response to first-line therapy, and tumor side in metastatic colon cancer patients

Polymorphisms	Dichotomization	Response to first-line therapy		*P* at χ^2 test	Tumor side		*P* at χ^2 test
		No DC (21 pts)	DC (78 pts)		Right	Left	
CDKN1B p.V109G	vs WT	11 vs 10	47 vs 31	0.5176	9 vs 21	32 vs 37	0.1304
CDKN2A p.A148T	vs WT	3 vs 18	7 vs 71	0.4757	0 vs 30	10 vs 59	0.0287
EML4 p.K409R	vs WT	10 vs 11	39 vs 39	0.8472	15 vs 15	34 vs 35	0.9474
HNF1A p.I27L	vs WT	21 vs 9	52 vs 26	0.4201	19 vs 11	45 vs 24	0.8577
HNF1A p.S487N	vs WT	9 vs 12	43 vs 35	0.3200	14 vs 16	38 vs 31	0.4438
INSR p.A2G	vs WT	13 vs 8	52 vs 26	0.6849	17 vs 13	48 vs 21	0.2165
INSR p.V975M	vs WT	0 vs 21	6 vs 72	0.1920	1 vs 29	5 vs 64	0.4556
IRS1 p.G971R	vs WT	3 vs 18	13 vs 65	0.7935	3 vs 27	13 vs 56	0.2745
IRS2 p.G1057D	vs WT	12 vs 9	41 vs 37	0.7102	16 vs 14	37 vs 32	0.9789
TCF7L2 p.P370R	vs WT	2 vs 19	3 vs 75	0.2941	2 vs 28	3 vs 66	0.6300
Coexistence of more than 3 polymorphisms	vs ≤3	17 vs 4	66 vs 12	0.6872	25 vs 5	58 vs 11	0.9286

Supplementary figures

Figure S1

Iconography of network visualization in Phenolyzer to depict interactional contexts.

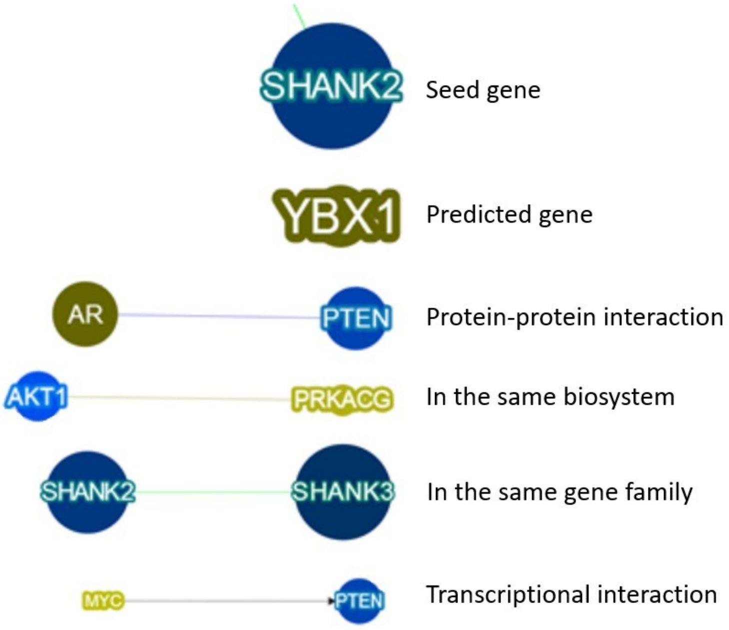

Supplement: Supplementary file 1 — DATA S1. Supporting information. [file IJC-157-1853-s003.pdf]
